# Comparative Evaluation of the Effect of Implant Crown Materials on Implant Components and Bone Stress Distribution: A 3D Finite Element Analysis

**DOI:** 10.1155/2023/1896475

**Published:** 2023-12-14

**Authors:** Kimia Nokar, Faezeh Atri, Saied Nokar

**Affiliations:** ^1^School of Dentistry, Tehran University of Medical Sciences, Tehran, Iran; ^2^Department of Prosthodontics, School of Dentistry, Craniomaxillofacial Research Center, Tehran University of Medical Sciences, Tehran, Iran; ^3^Department of Prosthodontics, School of Dentistry, Tehran University of Medical Sciences, Tehran, Iran

## Abstract

**Background:**

Choosing implant crown materials for restoration remains challenging in clinical practice. This study assesses the impact of all-ceramic restoration instead of porcelain-fused-to-metal (PFM) restoration on the stress distribution within implant components and the surrounding bone.

**Methods:**

Four 3D models of a mandibular second premolar were meticulously prepared. The study groups comprised zirconia, lithium disilicate, and zirconia lithium silicate monolithic ceramic crowns cemented onto a zirconia hybrid abutment. A PFM crown cemented onto a cementable abutment was chosen as the control group. A total vertical load of 583 N was applied to the occlusal contact areas. Stress distribution within the crown and implant components was analyzed using von Mises stress analysis. Principal stress analyses were employed to assess stress distribution in the peripheral bone.

**Results:**

The PFM model exhibited the highest von Mises stress values for both the implant (428.7 MPa) and crown (79.7 MPa) compared to the other models. The all-ceramic models displayed the highest maximum von Mises stress within the abutment, approximately 335 MPa, compared to the PFM model. von Mises stresses of the abutment and implant in the all-ceramic models were 69% higher and 20% lower, respectively, than those in the PFM model. Screw stresses were relatively consistent across all groups. Principal stresses in spongy bone and minimum principal stress in cortical bone were consistent across all models.

**Conclusions:**

All-ceramic restoration with a hybrid abutment, as opposed to traditional PFM restoration with a cementable abutment, does not adversely affect the implant and abutment screw and reduces crown stresses. Stresses within hybrid abutments were notably higher than those within cementable abutments. Spongy bone stresses remained unaffected by the type of crown or abutment.

## 1. Introduction

The emergence of CAD/CAM technology and high-strength dental ceramics has increased patient demand for replacing old metal–ceramic restorations with esthetic all-ceramic alternatives [1]. While insufficient compatibility was the primary obstacle preventing the use of some all-ceramic materials, zirconia and lithium disilicate ceramics have recently seen a surge in demand. Restorations constructed from these materials and cemented onto titanium or zirconia hybrid abutments have demonstrated clinical success and efficacy [2].

The hybrid abutment, combining the strength of titanium with the esthetics of ceramics, comprises two main components: a customized ceramic mesostructure and a titanium adhesive base [3]. The ceramic part is typically milled from a presintered block with the connector component. After sintering, the mesostructure is ready to be cemented onto the titanium adhesive base [2]. Surface treatment for the titanium base involves sandblasting with aluminum oxide to enhance surface roughness, followed by applying a metal primer [4]. These pieces are then assembled to form the hybrid abutment.

Several studies, including those conducted by Vazouras et al. [5], Adolfi et al. [6], and Alqarawi [7], have suggested that the use of hybrid abutments is a viable method for achieving esthetic outcomes in single implants, especially for anterior teeth. Moreover, the advantages of employing hybrid abutments over one-piece zirconia abutments include a reduced risk of screw loosening and tensile stress on the ceramic component. These abutments use a titanium-to-titanium interface to safeguard implant geometry and structures [2].

While ceramics are biocompatible and esthetic, their durability and fracture resistance must meet the criteria to replace conventional metal–ceramic crowns [8, 9]. Findings from a laboratory study comparing the fracture resistance of zirconia and lithium disilicate monolithic crowns, which were cemented onto zirconia hybrid abutments, indicated that these materials exhibit resistance to molar masticatory forces and yield satisfactory results [10]. However, the use of all-ceramic implant restorations with hybrid abutments in the posterior region remains debatable due to the lack of evidence [11–13].

Furthermore, it is crucial to consider the potential impact of restoration materials on bone morphology and surrounding tissue, as some studies have suggested that the type of material used can have adverse effects in these areas [14, 15].

Finite element analysis is an essential tool that enables us to reliably assess stress values and distribution within components, providing valuable insights into stress patterns in implant components and surrounding tissue [16]. Although these data may not be readily accessible in a clinical setting, they can be invaluable in identifying potential issues and developing practical solutions [17].

Excessive stress in implant and prosthetic components can lead to abutment screw loosening or fracture, loss of crown retention, and fracture of ceramic abutments or all-ceramic crowns. These complications can be avoided by controlling stresses within implant restoration structures [18, 19].

Regarding bone, finite element studies have indicated that factors such as the implant's position in the bone, the thickness of cortical bone, implant prosthetic platform angle, and the type of connection between implant and abutment influence the mechanical behavior of bone [20–22]. In contrast, the crown material generally has a minor influence at the bone level [20].

Based on the available information, there is a lack of evidence concerning the effect of all-ceramic restorations versus metal–ceramic restorations on implant component stress. Today, porcelain fuzed to metal (PFM) is considered the gold standard for implant-supported restorations [23].

Therefore, the present study aims to compare the stress on implant and bone components resulting from a single PFM crown on a cementable abutment as a control, and three types of CAD/CAM ceramic crowns (zirconia, zirconia lithium silicate, and lithium disilicate) cemented onto hybrid abutments using finite element analysis. The hypothesis is that different crown materials and abutment designs do not significantly affect the stresses on implant components and surrounding bone.

## 2. Materials and Methods

For modeling, the following components were scanned using an industrial scanner (Comet L3D; Carl Zeiss, Neubeuern, Germany): a bone-level implant with a diameter of 4.1 mm and a length of 12 mm (RC—Regular CrossFit; Straumann AG), a titanium abutment (RC Cementable Abutment-D 5 mm, GH 2 mm, AH 5.5 mm, Ti) along with its screw for PFM restoration, and a titanium base abutment (RC Variobase® for Crown—incl. screw, D 4.5 mm, AH 3.5 mm, GH 2 mm, TAN) along with its screw for CAD/CAM restorations. STL data from each component were imported into 3D simulation software, Catia v5-21 (Dassault System, Simulia Corp, USA), and 3D models were generated.

**Figure 1:**
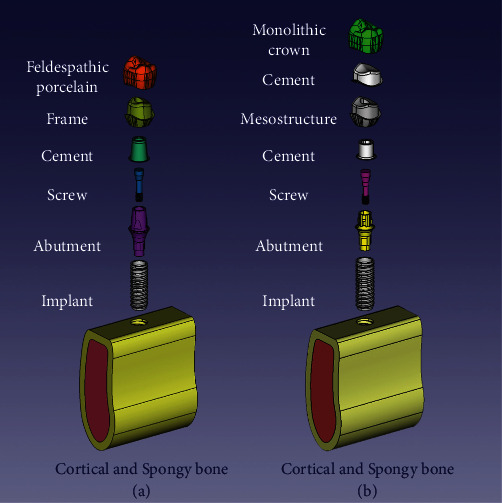
Illustration of the models: (a) PFM model; (b) all-ceramic model.

The 3D models were analyzed based on two variables: abutment type and restoration material, categorized as follows: (1) PFM: Cementable abutment with PFM crown, (2) Zr: Variobase abutment, zirconia mesostructure, and monolithic zirconia crown, (3) LD: Variobase abutment, zirconia mesostructure, and monolithic lithium disilicate crown, (4) ZLS: Variobase abutment, zirconia mesostructure, and monolithic zirconium lithium silicate crown (Figure 1).

In all-ceramic models, a zirconia customized mesostructure (5.5 mm height, 7 mm buccolingual, 5 mm mesiodistal width, and 2 mm lingual, 1 mm buccal, 3 mm mesial, and distal collar) was designed on the Variobase abutment. In the PFM model, a cementable abutment was used to design a PFM restoration with Cr─Co alloy and feldspathic porcelain. Subsequently, a crown for a mandibular second premolar (9 mm buccolingual and 7 mm mesiodistal) was designed for all models [24]. All monolithic ceramic crowns were designed with an axial wall thickness of 1 mm and an occlusal thickness of 1.5 mm (according to the manufacturer's recommendation). The thickness of the resin cement layer was set at 100 *μ*m in all groups [25].

The mandibular bone (type II) in the lower second premolar area was modeled with a height of 28.9 mm, a width of 17.43 mm, and a thickness of 13.6 mm, with 2 mm of cortical bone covering the spongy core [26]. It was assumed that osseointegration between the implant and bone was 100%, and the presence of gums was not considered in the models.

**Figure 2:**
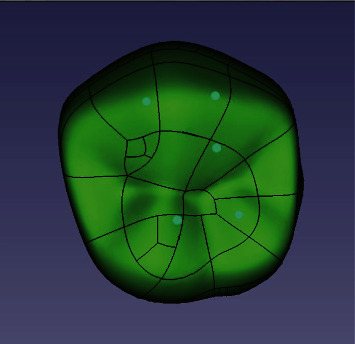
Spots show the location of vertical loads.

Meshing and stress distribution analysis was carried out using Abaqus 6.12 software. All materials were assumed to be homogeneous, isotropic, and linearly elastic. The structures were meshed with tetrahedral elements. The PFM model consisted of 161,617 nodes and 100,982 elements in total. Each all-ceramic model comprised 168,368 nodes and 104,926 elements in total. A preload torque of 35 Ncm was applied to the screw per the manufacturer's instructions. In each model, a force of 583 N (average chewing force in premolars) [27] was applied as a three-point contact in the slopes of the functional cusp and the depth of the fossa along the longitudinal axis of the implant (Figure 2) [28]. Young's modulus and Poisson's ratio for each material used were assigned in Table 1.

von Mises stress analysis was utilized to determine the stress distribution in implant components and the crown (specifically in the cervical third region). In contrast, maximum and minimum principal stress analyses were employed to assess the stress distribution in cortical and spongy bone structures.

## 3. Results

### 3.1. von Mises Stress Distribution

**Figure 3:**
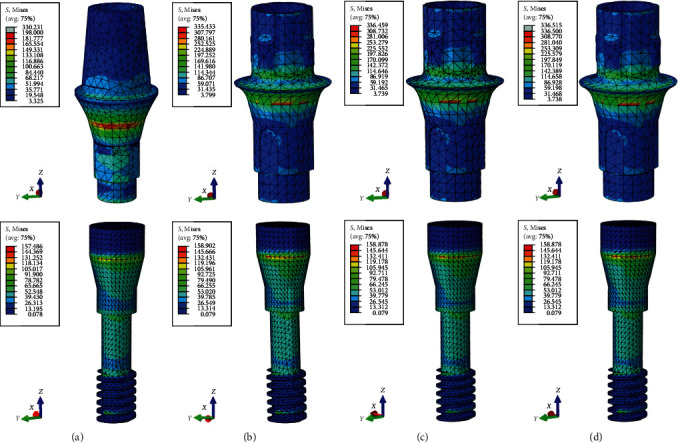
Location of maximum von Mises stresses of abutment and screw: (a) PFM; (b) Zr; (c) LD; (d) ZLS.

All model elements exhibited similar locations of von Mises stress concentration, specifically. All models' maximum von Mises stresses in the crown and cement were situated along the mid-buccal to distal cervical margins. In the all-ceramic models, the zirconia mesostructure displayed von Mises stress accumulation in the distal cervical margin. The highest stress concentrations in the abutments and their inner screws were found at the morse taper junction between the abutment and the implant, as well as in the shank area of the screws, respectively (see Figure 3). In all models, the von Mises stresses peaked at the implant's collar and the region where it contacts the crystal bone.

### 3.2. von Mises Stress Values

**Figure 4:**
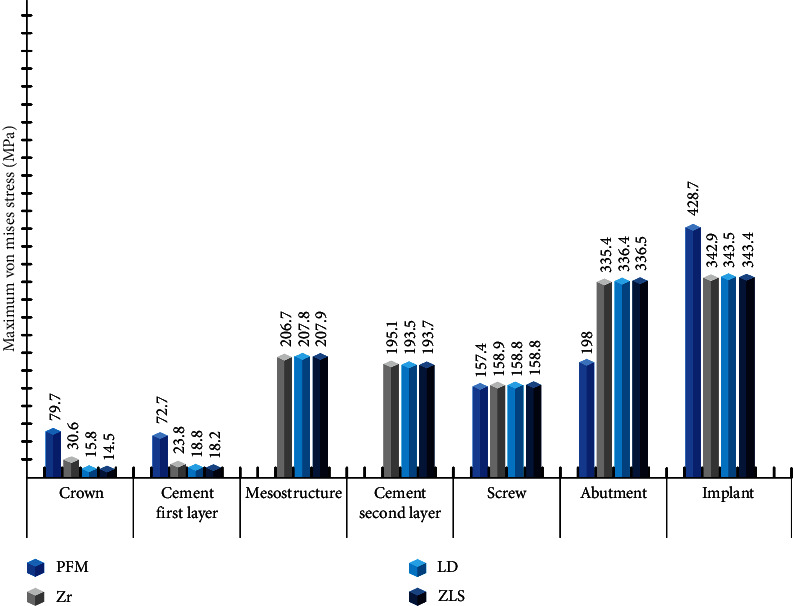
The maximum von Mises stress values in implant and restoration components (MPa).

The maximum von Mises stress values for the Zr, LD, ZLS, and PFM models are depicted in Figure 4. In the PFM model, the maximum von Mises stress for the Cr─Co metal frame and its porcelain layer were reported as 79.7 MPa and 16.35 MPa, respectively. Among all ceramic models, the metal framework displayed the highest maximum von Mises stress compared to the monolithic crown. The PFM cementable abutment exhibited a significantly lower stress value than the all-ceramic models' Variobase abutment, with a 69% reduction. The maximum von Mises stress of the implant in the PFM model was reported to be 20% higher than that in all ceramic models. Ceramic models exhibited nearly identical maximum von Mises stress values in all elements except for the crown, which was observed to be nearly twice as high as the others in the zirconia model.

### 3.3. Principal Stresses in the Bone

**Figure 5:**
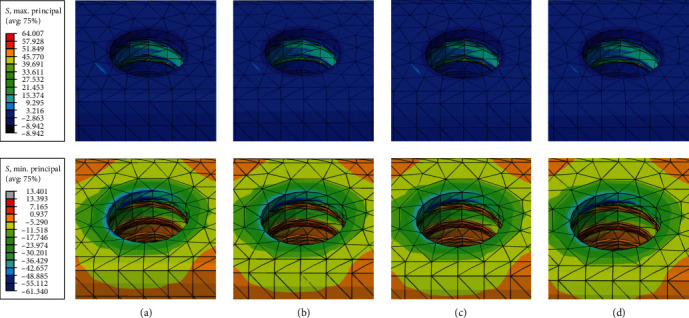
Location of maximum and minimum principal stresses in cortical and spongy bone (MPa): (a) PFM; (b) Zr; (c) LD; (d) ZLS.

**Figure 6:**
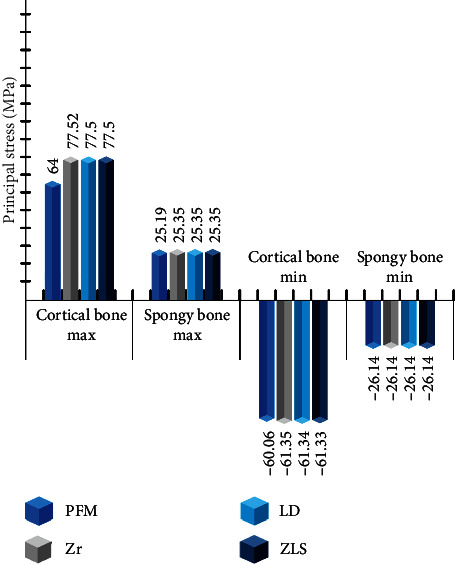
Maximum and minimum principal stress values in cortical and spongy bone (MPa).

The locations of maximum and minimum principal stresses in the cortical and cancellous bone were consistent across all models, primarily in the bone crest area on the distal and lingual sides (refer to Figures 5 and 6). All models demonstrated similar principal stress values in bone, except for the maximum principal stresses in the cortical bone, which were approximately 21% higher in the all-ceramic models, measuring 13.5 MPa.

## 4. Discussion

This finite element study compared stress patterns and values in all-ceramic models versus the PFM model. Finite element analysis results are commonly expressed in von Mises stress and maximum and minimum principal stresses. von Mises stress analysis is typically suited for ductile materials, such as titanium, that display equal compressive and tensile strength [17]. In contrast, the maximum principal stress is a more appropriate indicator for brittle materials like ceramics and bone since it distinguishes between tensile and compressive stresses through positive and negative signs [36].

Most previous research studies have employed von Mises stress as the primary analysis criterion. This study used maximum and minimum principal stress analyses for bone, and von Mises stress analysis was used for implant and prosthetic components.

The stress values observed in the restorative crowns varied due to their different moduli of elasticity. The results suggest a positive correlation between the crown material's modulus of elasticity and stress accumulation within the crown; therefore, the hypothesis was rejected.

Present findings partially support the conclusions drawn in previous studies [37, 38], which suggested that utilizing a more rigid crown would increase stress within its structure while reducing stress in the solid abutment composed of zirconia or titanium. While the current study demonstrates a similar relationship between the crown's modulus of elasticity and its stress distribution, it does not corroborate the latter part of the statement above, as consistent stresses were observed in hybrid abutments irrespective of the crown material used. These results align with a study by Tribst et al. [39], which also used hybrid abutments.

Moreover, the clinician should consider that although the stress accumulation in the crown is higher in the PFM and Zr models, those are naturally more resistant to fracture. In all mentioned materials, including different types of ceramic and titanium, maximum von Mises stress values were below the material's yield strength [40–42]. However, it cannot be claimed that structure failure will not occur. As it is known, zirconia goes through low-temperature degradation (aging) due to stresses and being in a wet environment at low temperatures [43]. With aging, the tetragonal phase transforms into a monolithic phase, which has less flexural strength and less desirable mechanical properties. These changes may lead to failure in the future [44].

Systematic reviews [18, 19, 45, 46] evaluating the clinical performance of metal–ceramic and all-ceramic restorations have indicated that chipping of ceramic veneers is a common issue in metal–ceramic restorations. Research suggests that fracture is less likely to occur in monolithic zirconia restorations; however, if feldspathic ceramic is used for veneering the monolithic zirconia, the chipping rate could still be similar to that of metal–ceramic restorations. In conclusion, using all-ceramic monolithic restoration seems to be an excellent alternative to metal–ceramic restoration. In addition, a study by Skjold et al. [47] investigated failed monolithic and bi-layered zirconia ceramic crowns obtained from dental practices to evaluate their fracture modes. The study found that the primary fractures were observed in the margins of the crowns.

The stress levels in Variobase abutments were similar and much higher than those in cementable abutments. However, some in vitro studies show hybrid abutments could function as effectively as titanium abutments [10, 48, 49]. This discrepancy appears to be attributed to the disparity in the height of the cementable abutment (GH 2 mm, AH 5.5 mm) compared to the Variobase abutment (AH 2 mm, GH 3.5 mm), which leads to increased leverage forces. Additionally, the difference in the geometric shape of these two abutments can impact stress distribution and magnitude.

The stress values in the zirconia mesostructure were relatively consistent across all models. This implies that using different ceramic materials for the crown will not affect the stress levels in hybrid abutments.

Stresses in the screw were similar in magnitude and location (in the shank area) in all models, irrespective of crown or abutment design and material. The likely reason is the region's Morse taper design and torque application. In all-ceramic crowns cemented on hybrid abutments, the abutment screw may be the sole vulnerability regarding fatigue strength [50]. It can be tentatively concluded that all-ceramic restoration will not increase the risk of screw loosening compared to the PFM model. An alternative to avoid screw-related complications is using abutments without screws; however, a finite element study conducted by Epifania et al. [37] compared different locking systems with or without screws, and it concluded that the absence of the screw led to an increase in stress within the abutment and implant.

In all-ceramic models compared to the PFM model, von Mises stress values in the abutment and implant were higher and lower, respectively. In all ceramic models, the abutment absorbs a portion of the force, reducing stress accumulation in the implant.

Considering factors influencing stress distribution in bone, studies have recommended using a platform-switched internal connection between the implant and abutment [20, 21], placing the implant at bone level [20], and selecting cases with a thicker cortical bone [22] to mitigate stress on the bone and surrounding tissue. This study employed an internal connection with all models' Morse taper platform-switched design.

Various finite element studies have indicated that using different materials for crowns and abutments within the same design yields similar stress values in bone [38, 51], and the results of this study are in concordance with prior research, as stress values were approximately uniform among all-ceramic models. In contrast, the maximum principal stress in the cortical bone of the PFM model was approximately 20% lower than that of all-ceramic models, suggesting that all-ceramic designs generate more stress in the cortical bone at the crest area.

The absence of dynamic loading and thermal effects constitutes the limitations of the present study. Therefore, further research is warranted to assess the clinical efficacy of these materials.

## 5. Conclusions

Within the scope of this study, it has been determined that utilizing all-ceramic restoration in conjunction with a hybrid abutment, as opposed to employing a conventional PFM restoration with a cementable abutment, does not yield any adverse effects on the implant or abutment screw. Furthermore, it is found to alleviate the stresses on the crown. It is worth noting that the stresses experienced in Zirconia hybrid abutments are markedly higher when compared to those in cementable abutments.

The stresses on spongy bone are not influenced by the type of crown or abutment used. However, the maximum principal stresses within the cortical bone of the PFM model were lower than all ceramic designs.

Subsequent research endeavors should be conducted within a clinical context to explore these findings further.

## Figures and Tables

**Table 1:** Properties of used material.

Material	Young modulus (GPa)	Poisson's ratio	Reference
Cortical bone	13.7	0.30	[29]
Cancellous bone	1.37	0.30	[29]
Titanium implant	117	0.33	[30]
Titanium abutment	117	0.33	[30]
Zirconia (Lava; 3M ESPE)	210	0.3	[31]
Lithium disilicate (Vita Suprinity)	65.6	0.23	[32]
Zirconia lithium silicate (Celtra Duo)	61	0.30	[33]
Dual polymerized resin cement	18.6	0.28	[34]
Metal alloy (Cr Co)	220	0.30	[35]
Feldspathic porcelain	48.7	0.23	[35]

## Data Availability

All the data generated or analyzed during this study are included in the article.
